# Towards bottom-up nanopatterning of Prussian blue analogues

**DOI:** 10.3762/bjnano.5.204

**Published:** 2014-10-31

**Authors:** Virgile Trannoy, Marco Faustini, David Grosso, Sandra Mazerat, François Brisset, Alexandre Dazzi, Anne Bleuzen

**Affiliations:** 1 Institut de Chimie Moléculaire et des Matériaux d’Orsay,UMR CNRS 8182, Université Paris-Sud, 15 rue Georges Clémenceau, 91405 Orsay Cedex, France; 2 Laboratoire de Chimie de la Matière Condensée de Paris, Université Pierre et Marie Curie-Paris 6 and CNRS Collège de France, 11 place Berthelot 75231 Paris, France; 3 Laboratoire de Chimie Physique, UMR CNRS 8000, Université Paris-Sud, 15 avenue Jen Perrin, 91405 Orsay Cedex, France

**Keywords:** nanopatterning, nanoperforated oxide monolayer, Prussian blue analogues

## Abstract

Ordered nanoperforated TiO_2_ monolayers fabricated through sol–gel chemistry were used to grow isolated particles of Prussian blue analogues (PBA). The elaboration of the TiO_2_/CoFe PBA nanocomposites involves five steps. The samples were characterized by scanning electron microscopy (SEM), atomic force microscopy (AFM), infrared spectroscopy and X-ray photoelectron spectroscopy (XPS) all along the synthesis process. Selected physico-chemical parameters have been varied in order to determine the key steps of the synthesis process and to optimize it. This study is an important step towards the full control of the fabrication process.

## Introduction

The development of methods to place nanoparticles into spatially well-defined, ordered arrays is one challenging aspect of nanotechnology. This is usually achieved by using top-down approaches, implementing optical and electron beam lithography. Here, we explore the possibilities of elaborating nanopatterned surfaces by a pure bottom-up approach.

The nanopatterned surfaces are mainly built from molecular precursors in solution through a succession of chemical steps. The advantages of this approach are very low fabrication costs, and easy adaptability of the fabrication process to the industrial scale. Our synthesis process is based on two main chemistries. The positioning of the functional objects and their isolation from each other is realized thanks to the exceptional processing flexibility inherent to sol–gel chemistry combined with organic templating agents. Coordination chemistry allowing for the controlled assembly of a large variety of transition metal building units is preferred to build the functional compound.

Prussian blue analogs (PBAs) are interesting for the design of bistable compounds for two reasons. Firstly, some of them are molecular magnets with an ordering magnetic temperature that can exceed room temperature [1–2]. Secondly, some PBAs exhibit a photomagnetic effect [3–4]. Thus, in some CoFe Prussian blue analogs, an irradiation in the visible range transforms a diamagnetic state into a ferrimagnetic state with a long life time. This photomagnetic effect is interesting for high-density storage since the property of bistability is intrinsically molecular and therefore persists up to molecular scale [5–8]. These coordination polymers are obtained by a reaction between hexacyanometalates and hydrated cations of the transition metal series in aqueous solution. The resulting solid exhibits the well-known face centered cubic structure of Prussian blue [9].

The sol–gel process is a method for producing metal oxides from small molecules via inorganic polymerization reactions in solution. The sol–gel transition allows one to obtain the oxide in any desired shape including films, fibers, monolithes [10]. Furthermore, the addition of self-assembling liquid crystalline templates to the sol can lead to the ordered nanostructuration of the oxide matrix [11]. Thus, nanoperforated thin layers_,_ exhibiting hexagonal arrays of nanoperforations aligned perpendicular to the surface of the film surface have been developed [12–13]. These nanoperforated films are particularly appealing to organize isolated bistable dots on a solid surface [14–15].

The elaboration of the oxide/Prussian blue analogue nanocomposite involves five main steps, which have been described elsewhere for the fabrication of nanometer-scale patterns of photo-switchable PBA [15]. The substrate is first covered with a gold layer through sputtering, which will allow for the chemical differentiation of the surfaces and therefore their selective functionalization in the following. The second step is the deposition through dip-coating of an ethanolic solution of titanium molecular species containing block copolymers to obtain an ordered nanostructured organic–inorganic hybrid layer. The third step is a thermal treatment, which induces the decomposition of the organic part and the crystallization of the titanium dioxide leading to the nanoperforated layer. The fourth step is the selective functionalization of the surfaces to localize the PBA growth within the perforations while avoiding its formation outside. The last step is the PBA growth by a layer-by-layer directed assembly approach inspired from methods already implemented for the synthesis of PBA thin films [16–18].

Here, we study the impact of each step on the structure of the nanocomposite. We show that the first gold layer can undergo some changes in the course of the fabrication process and has to be optimized in order to obtain well-ordered nanoperforated oxide layers. We also show that the density of filled perforations strongly depends on physico-chemical parameters used for the PBA growth step, which turns out to be the trickiest step of the elaboration process.

## Experimental

### Synthesis of the TiO_2_/PBA nanocomposite

The five steps involved in the fabrication of the PBA/TiO_2_ nanocomposites are the following [15]: In a first step, the silicon substrate is covered by a gold layer by sputtering under vacuum in a sputter coater (Quorum, Q150T ES) for use in scanning electron microscopy. This gold layer will allow for the chemical differentiation of the surfaces in the following. Layers having thicknesses of 10 nm, 20 nm and 50 nm were deposited under an electric current of 30 mA during calibrated times (30 s, 60 s and 150 s). The three samples resulting from this first step and corresponding to the different thicknesses are called **Au10**, **Au20** and **Au50** in the following (see below in Table 1).

The second step is the deposition by dip-coating of an ethanolic solution of titanium molecular species containing block copolymers micelles that after evaporation are self-assembled to obtain an ordered nanostructured organic–inorganic hybrid layer. The solution for dip-coating was prepared by dissolving 37.5 mg of polybutadiene-*block*-poly(ethylene oxide) (*M*_w_(PB) = 22000 g·mol^−1^, *M*_w_(PEO) = 15500 g·mol^−1^) in 9.85 g of EtOH and 0.5 g of H_2_O at 70 °C for 2 h. It was added then 0.27 g of an ethanolic solution of TiCl_4_ (molar ratio 1:5) [12,19]. The deposition of the film was performed under controlled conditions of temperature of the chamber (80 °C), of ascent speed (2 mm/s) and of humidity rate (<2%).

**Scheme 1:**
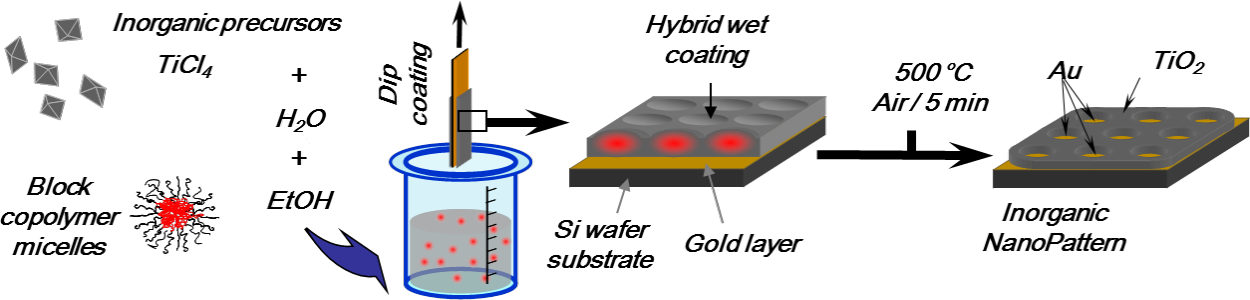
Fabrication of the nanoperforated TiO_2_ layer (steps 2 and 3).

The third step is a thermal treatment under an IR-lamp at 450 °C over 5 min, which results in the decomposition of the organic part and the crystallization of the titanium dioxide leading to the nanoperforated layer (ca. 15 nm) with homogeneous and ordered holes (50 nm in diameter) giving access to the gold layer underneath (Scheme 1) [15]. The three samples resulting from this third step and corresponding to the three different thicknesses of the gold layer are called **Au10NC**, **Au20NC** and **Au50NC** in the following (see below in Table 1).

**Scheme 2:**
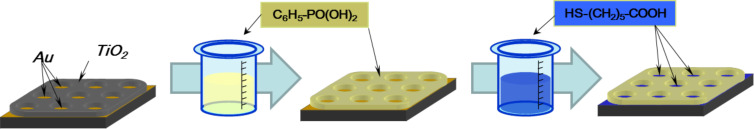
Selective functionalization of the TiO_2_ and gold surfaces (step 4).

The fourth step is the selective functionalization of the surfaces. In order to prevent the formation of PBA outside the nanoperforations, the TiO_2_ surface was passivated by grafting with hydrophobic groups. The sample was placed in a solution of phenylphosphonic acid (3·10^−3^ M) in an ethanol/water mixture (3:1 EtOH/H_2_O) for 12 h. The film was rinsed with EtOH and acetone and allowed to dry at 120 °C for 2 h. Then, an anchoring layer for PBA was grafted onto the gold bottom of the nanoperforations by dipping the substrate in an ethanolic solution of mercaptohexanoic acid (3·10^−3^ M), 4-mercaptopyridine (3·10^−3^ M) or 4-aminothiophenol (3·10^−3^ M) for 12 h. Mercaptohexanoic acid, 4-mercaptopyridine or 4-aminothiophenol are abbreviated as MHA, 4-MPy and 4-ATP, respectively, in the following. The film was rinsed with EtOH and water. Scheme 2 shows the successive functionalization steps of the film in the case of the MHA anchorage [20–21].

**Scheme 3:**
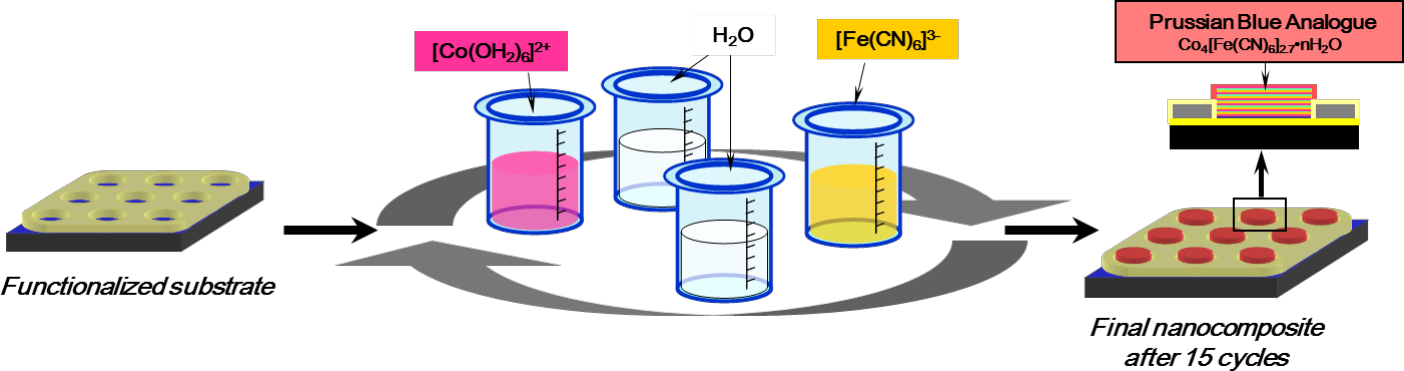
Layer-by-layer PBA growth (step 5).

The last step is the PBA growth through a layer-by-layer approach consisting of successive immersions of the functionalized substrate in PBA precursor solutions. The substrate was first immersed in a hexaaquacobalt(II) solution for 12 h. Then, it was successively immersed for 2 min in a 3·10^−3^ M [Fe(CN)_6_]^3−^ aqueous solution and in a 10^−1^ M [Co(H_2_O)_6_]^2+^ aqueous solution. The substrate was carefully rinsed with water after each immersion. The sample was dipped 15 times in both solutions (Scheme 3) [15].

The same protocol was carried out while replacing the water solvent with i) butanol for the Co^2+^ solution and ethanol/water (1:1 EtOH/H_2_O) for the [Fe(CN)_6_]^3−^ solution and ii) ethanol/water (1:1 EtOH/H_2_O) for the Co^2+^ solution and for the [Fe(CN)_6_]^3−^ solution. The name and the conditions used for the preparation of the samples resulting from this fifth step are gathered in Table 1 (**NC01**–**NC05**).

**Table 1:** Conditions used for the preparation of the different samples.

name of the sample	thickness of the gold layer/nm	TiO_2_ deposition	gold functionalization	solvent or mixture of solvents for the PBA precursor solution	
				**Co^2+^**	**[Fe(CN)_6_]^3−^**

**Au10**	10	—	—	—	—
**Au20**	20	—	—	—	—
**Au50**	50	—	—	—	—
**Au10NC**	10	×	—	—	—
**Au20NC**	20	×	—	—	—
**Au50NC**	50	×	—	—	—
**NC01**	20	×	MHA	H_2_O	H_2_O
**NC02**	20	×	4-ATP	H_2_O	H_2_O
**NC03**	20	×	4-MPy	H_2_O	H_2_O
**NC04**	20	×	MHA	BuOH	EtOH/H_2_O = 1:1
**NC05**	20	×	MHA	EtOH/H_2_O = 1:1	EtOH/H_2_O = 1:1

### Materials characterization

SEM images were obtained by using a field emission gun scanning electron microscope (FEG-SEM Zeiss Sigma HD microscope) equipped with an in-lens detector working at 1 kV and at a short working distance (WD) equal to 3.3 mm for the TiO_2_ thin film images and equipped with a secondary electron detector (SE) working at 1 kV and at a short working distance (WD) equal to 3.8 mm for the TiO_2_/PBA nanocomposite images. Tapping mode topography and phase imaging was accomplished by using an Innova AFM (Bruker) with NanoDrive v8.02 software. Tapping mode images were acquired by using silicon tips from Nanosensors (PPP NCSTR) with a resonance frequency ranging between 76 and 263 kHz. Images were processed by using WsXM software. Fourier transform-infrared (FTIR) spectra were collected in the attenuated total reflection (ATR) mode by using a Vertex 70 spectrometer with a germanium crystal. XPS spectra were collected on a SPECS (Phoibos MCD 150) X-ray photoelectron spectrometer, by using Mg Kα (*h*ν = 1253.6 eV) X-ray source having a 150 W (12 mA, 12.5 kV) electron beam power and a 7 × 20 mm spot size. The emissions of photoelectrons from the sample were analyzed at a takeoff angle of 90° under ultra-high vacuum conditions (1·10^−8^ Pa). High resolution spectra were collected at a pass energy of 10 eV for S 2p core XPS levels. No charge compensation was applied during acquisition.

## Results and Discussion

### The gold layer

**Figure 1:**
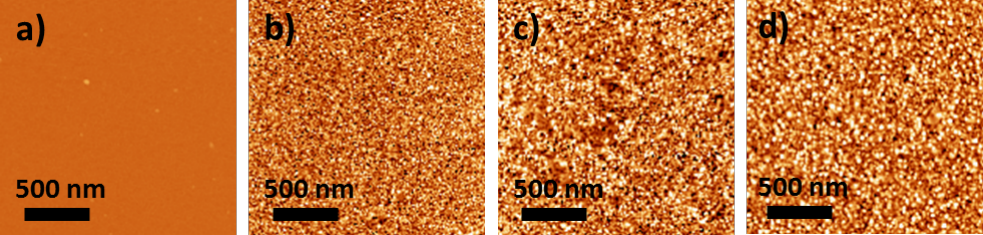
AFM images of a) the Si substrate b) **Au10**, c) **Au20** and d) **Au50**.

The silicon wafer and the samples **Au10**, **Au20** and **Au50** were studied by AFM. Representative AFM images are shown in Figure 1. After gold deposition, whatever the deposition time, the silicon substrate is completely and homogeneously covered by gold nanoparticles (as expected for sputtering deposition). But, the size of the particles depends on the deposition time. The gold layers obtained with 30 s, 60 s and 150 s deposition time are composed of gold nanoparticles of 10 nm, 15 nm and 40 nm in diameter, respectively. The roughness is 0.27 nm for the silicon substrate, 0.44 nm for the 10 nm thick gold layer, 0.48 nm for the 20 nm thick gold layer and 1.01 nm for the 50 nm thick one. After the gold deposition, the surfaces exhibit a small roughness and are therefore of good quality for the nanoperforated oxide layer deposition.

### The nanoperforated TiO_2_ monolayer

**Figure 2:**
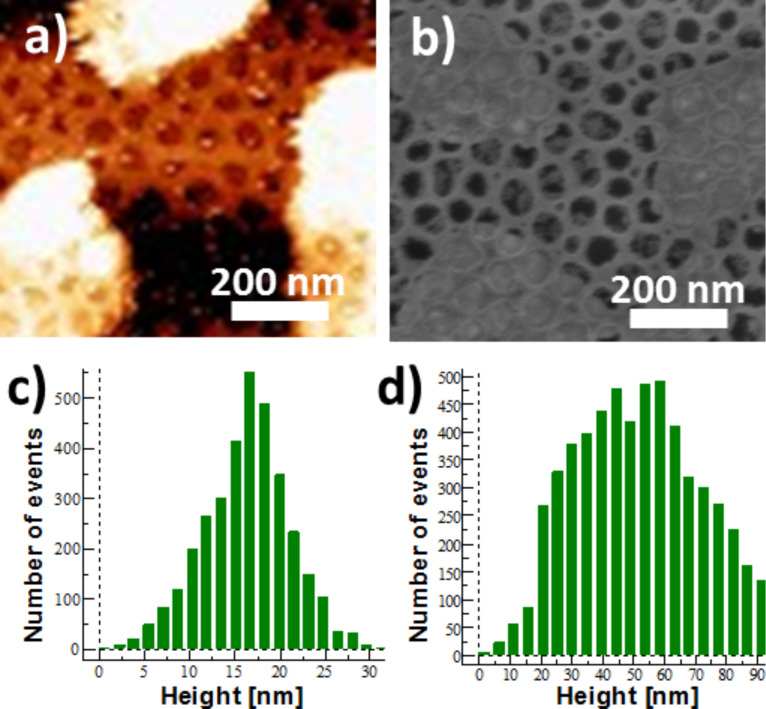
a) AFM and b) SEM images of the sample **Au10NC**. Depth distribution histogram in the c) dark and d) light areas.

The nanoperforated TiO_2_ monolayer deposited on the silicon wafer covered by a 10 nm thick gold layer (sample **Au10**) was studied by AFM and SEM. Representative AFM and SEM images are shown in Figure 2a and Figure 2b. Two different kinds of zones can be observed on the AFM and SEM pictures. Some lighter islands appear on the darker background. The fraction of the light areas can be estimated to be around 40%. The dark spots correspond to nanoperforations aligned perpendicularly to the surface. Their depth has been estimated to be 15 nm and their diameter to be 50 nm (Figure S1 in Supporting Information File 1). The distance between two adjacent nanoperforations is 80 nm. Whatever the area, light or dark, the 2D-hexagonal organization of the perforations is visible. This indicates that the nanoperforated oxide film covers the whole surface. The two kinds of zones have been assigned to areas with and without gold between the silicon substrate and the perforated oxide layer. Figure 2c and Figure 2d show the depth distribution histogram in the dark and light areas of the AFM image. This AFM study indicates that the bright zones have higher relief (up to 80 nm above the silicon wafer) with a wide height distribution. In contrast, the depth distribution histogram in dark areas shows a narrow distribution centered around 15 nm corresponding to the thickness of the TiO_2_ nanoperforated layer. This suggests a dewetting of the gold layer from the silicon surface to form gold droplets between the silicon wafer and the nanoperforated oxide layer corresponding to the light areas. The bottom of the nanoperforations in the dark areas would therefore be made of silicon rather than of gold. This hypothesis has been confirmed by energy dispersive X-ray analysis (EDX) performed in a bright and in a dark area. The results clearly show the presence of gold in the bright area, whereas this element is absent in the dark area (Figure S2 in Supporting Information File 1).

**Figure 3:**
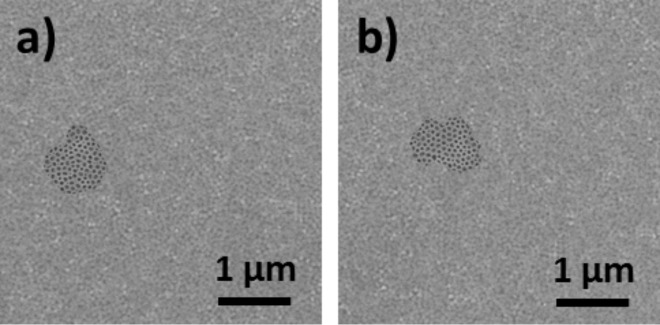
SEM micrographs of a) **Au20NC** and b) **Au50NC**.

**Figure 4:**
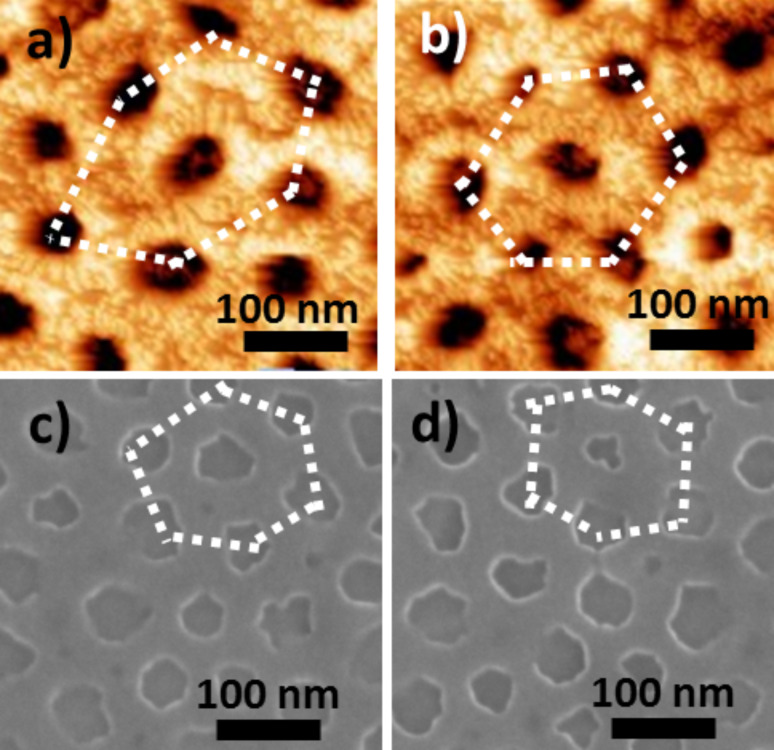
AFM images of a) **Au20NC**, b) **Au50NC** and SEM micrographs of c) **Au20NC** and d) **Au50NC**.

Nanoperforated TiO_2_ monolayers deposited on 20 nm and 50 nm thick gold layers (samples **Au20NC** and **Au50NC**) were also studied by AFM and SEM. Representative SEM images of **Au20NC** and **Au50NC** at a low magnification are shown in Figure 3, and AFM and SEM images with higher magnification are shown in Figure 4.

The SEM images of **Au20NC** and **Au50NC** show few dark islands of around 1 μm, standing out from the light background. As for the sample **Au10NC**, the 2D organization of the nanoperforations is visible over the whole surface. The minority of dark areas (less than 6% of the whole surface) are assigned to zones where a dewetting of the gold layer from the silicon substrate occurred. In contrast to sample **Au10NC**, the gold layer still covers a great majority of the surface after the formation of the oxide nanoperforated layer.

Figure 4, corresponding to a magnification of the majority of light areas, shows that the AFM and SEM images of the films obtained with the 20 nm and the 50 nm gold layers are comparable. They show dark spots corresponding to nanoperforations of same depth and of same diameter as those obtained with the 10 nm thick gold layer and exhibiting a 2D-hexagonal organization (shown by the dashed lines in Figure 4). Nevertheless, the degree of hexagonal order is lower for these films deposited on gold layers than for films directly deposited on the silicon substrate [19,22–24]. This loss of order is probably due to matter displacement during the thermal treatment, leading to an accumulation of gold in some regions of the sample and to a lack of gold in others. As a consequence, the TiO_2_ nanoperforated layer forms on an undulating gold surface, which results in a certain lowering of the degree of long-range order.

**Figure 5:**
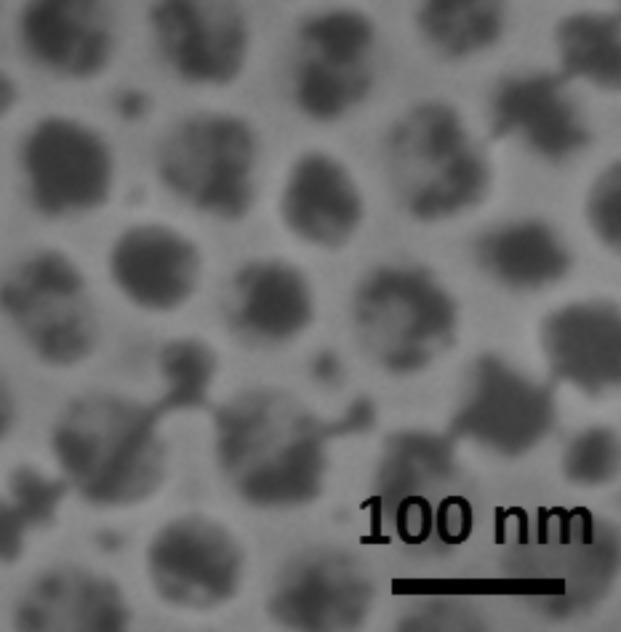
SEM image corresponding to a dark area of the sample **Au20NC**.

Whatever the sample, the AFM and SEM studies also revealed the presence of some perforations, the bottom of which seems not to be completely cleared of TiO_2_. This effect is more visible in areas without gold. A SEM image corresponding to such areas of sample **Au20NC** is shown in Figure 5. A light grey pellicle, indicating the presence of TiO_2_, partially covers the bottom of some perforations.

The non-homogeneous and non-planar surface exhibiting a different chemical nature of the bottom of the nanoperforations of the films deposited on a 10 nm thick gold layer, such as sample **Au10NC**, can be assigned to a dewetting of the gold layer from the silicon during the thermal treatment at 450 °C. This inhomogeneity of the surface led us to exclude samples with such gold layers for the study. The films synthesized with the 20 nm thick gold layer, such as sample **Au20C**, were used in the following without further optimization. Nevertheless, this study shows an unexpected behavior of the gold layer, which has to be optimized in the future.

### Functionalization of the Au and TiO_2_ surfaces

To localize the growth of PBA into the nanoperforations, a selective functionalization of the Au and TiO_2_ surfaces has been implemented. The oxide surface is rendered hydrophobic in order to prevent the adsorption of PBA precursors. The phosphonate function allows for the selective grafting of the phenyl group on TiO_2_ [20–21]. Then, MHA, 4-MPy or 4-ATP are used as coupling agents for anchoring a first layer of Co^2+^ ions at the bottom of the nanoperforations.

**Figure 6:**
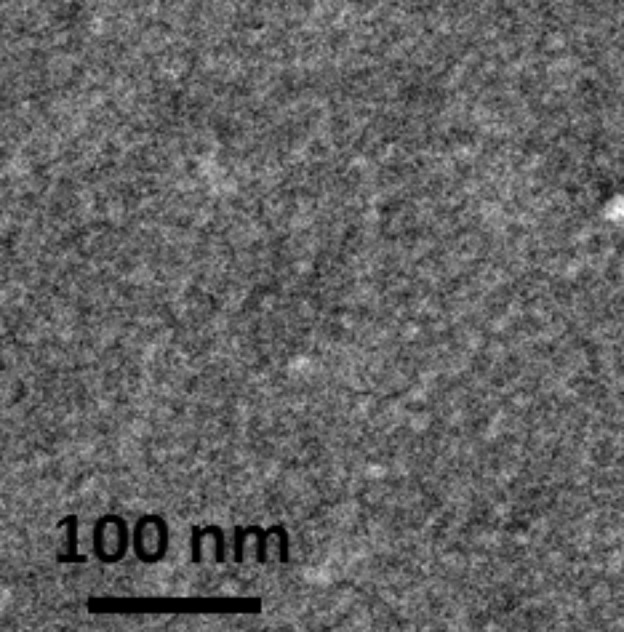
SEM image corresponding to CoFe PBA grown on the nanoperforated films without prior functionalization of TiO_2_.

To evaluate the efficiency of the TiO_2_ functionalization, this step was omitted while the functionalization with MHA and the growth of CoFe PBA in aqueous solution were performed. A representative SEM image of this sample is shown in Figure 6. The whole surface seems bumpy as if a crust constituted by very small PBA particles covered it and under which the nanoperforations are hardly visible. Without hydrophobization of TiO_2_, PBA precursors interact with the accessible oxide surface leading to the formation of PBA particles everywhere.

**Figure 7:**
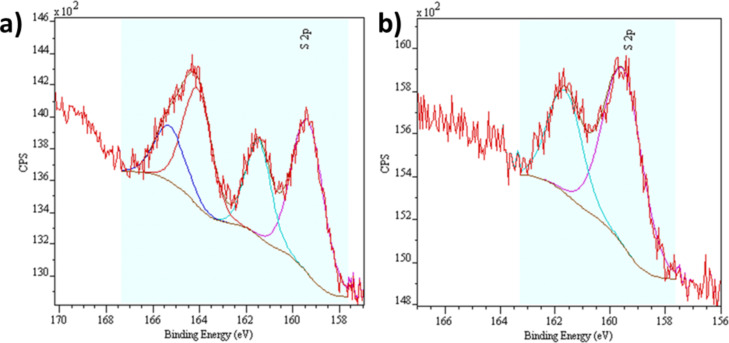
XPS spectra of a film immersed in MHA solution a) before and b) after rinsing with EtOH.

In order to verify the effectiveness of the gold functionalization through the MHA anchoring function at the bottom of the perforations, the presence of sulfur atoms at the surface of the sample was monitored by X-ray photoelectron spectroscopy (XPS) throughout the functionalization step. XPS was carried out right after removing the sample from the MHA solution and after rinsing with EtOH (Figure 7). Before rinsing (Figure 7a), the spectrum exhibit two doublets that can be assigned to a free thiol function and a thiolate species bound to the gold surface [25–26]. After rinsing, the band characteristic of the free function has disappeared (Figure 7b). But, the band corresponding to bound thiolate species still remains. The grafting of the gold surface with MHA therefore was successful.

The effect of the functionalized gold layer at the bottom of the perforations on the growth of PBA is nicely illustrated by a SEM image of the film exhibiting areas with and without gold after PBA growth. PBA particles are concentrated in the areas with gold at the bottom of the nanoperforations, whereas the areas without gold are almost completely free of PBA particles (Figure S3 in Supporting Information File 1).

### TiO_2_/PBA nanocomposites

#### Microscopy study

**Figure 8:**
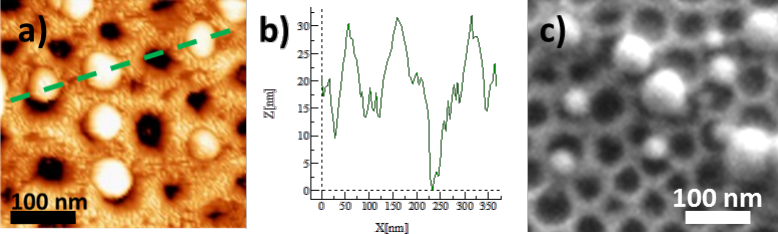
a) AFM image, b) height profile along the green dotted line on the AFM image and c) SEM image of **NC01**.

The TiO_2_/CoFe PBA nanocomposite **NC01** synthesized by using MHA as anchor and water as the solvent was studied by AFM and SEM. Representative AFM and SEM images of the sample **NC01** are shown in Figure 8a and Figure 8c. The height profile along the green dashed line passing through one hole and three particles on the AFM image is shown in Figure 8b.

The AFM and SEM images show the 2D-hexagonal arrangement of the nanoperforations and some light objects assigned to PBA particles localized on some of the nanoperforations (Figure 8a and Figure 8c). The shape of the particles is spherical or half-spherical. On the TEM image it is difficult to see if the particles are located in the pores or on the TiO_2_ grid. On the contrary, in the AFM image, the particles clearly seem to be located in the nanoperforations. Furthermore, the AFM height profile (Figure 8b) shows three maxima and one minimum corresponding to three particles and one perforation. The distance between these maxima and minimum fairly corresponds to the distance between adjacent nanoperforations of the TiO_2_ film before PBA growth (see above), which supports the location of the particles on the nanoperforations. Nevertheless, one cannot conclude whether or not the particles are anchored to the bottom of the perforations.

Each particle seems to be located on one perforation, but not all of the nanoperforations are filled. The average rate of perforations containing one PBA particle is around 15%. The localization of the PBA particles in the perforations indicates that the hydrophobic groups have successfully passivated the oxide surface. The partial loading of the perforations by PBA particles can be due to several reasons listed below:

- the presence of TiO_2_ at the bottom of the perforations, which have been rendered hydrophobic when the substrate has been immersed in phenylphosphonic acid, which prevents i) the grafting of the anchoring function for PBA growth and ii) the adsorption of any of the PBA precursors or PBA particles;

- a low yield of the complexation reaction of the Co^2+^ ions by the anchoring functions grafted onto the bottom of the nanoperforations;

- the occurrence of dissolution–recrystallization phenomena during the PBA growth step: during the successive immersions of the functionalized film, the particles or a few of the particles formed during the first cycles can dissolve and the released precursors reform new particles.

The size of the PBA particles ranges from 20 to 100 nm. The size of the smallest particles is of the order of magnitude expected for PBA particles formed by a layer-by-layer approach since 15 units of –Fe–CN–Co- (corresponding to 15 cycles) corresponds to approximately 8 nm. Therefore, a growth from Co ions anchored to the bottom of the perforations could result in half-spherical particles of 16 nm in diameter. The formation of particles of bigger size can be explained by the occurrence of dissolution–recrystallization phenomena.

**Figure 9:**
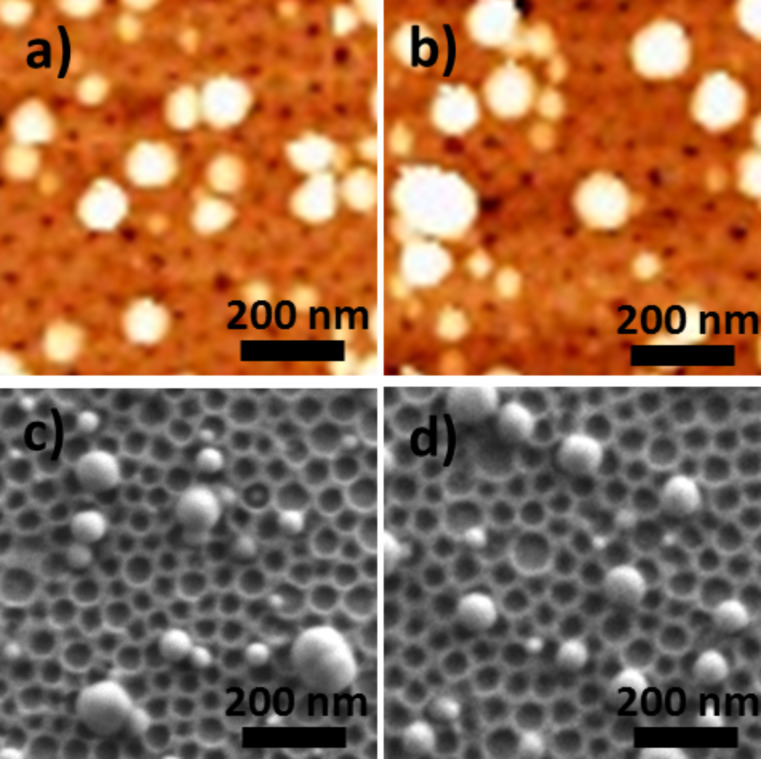
AFM images of a) **NC02**, b) **NC03** and SEM micrographs of c) **NC02**, d) **NC03**.

**Effect of the chemical nature of the anchoring function for PBA:** The TiO_2_/CoFe PBA nanocomposites **NC02** synthesized by using 4-MPy (Figure 9a and Figure 9c) and **NC03** synthesized by using 4-ATP (Figure 9b and Figure 9d) as anchoring functions while keeping water as the solvent were studied by AFM and SEM. Representative AFM and SEM images are shown Figure 9.

The AFM and SEM images are very similar to those obtained with MHA. They show the same spherical or half-spherical particles localized on the nanoperforations. The size of these PBA particles ranging from 20 to 80 nm in diameter is comparable to that of the previous sample. As before, around 15% of the nanoperforations are filled with PBA particles. The chemical nature of the complexing agent for the transition metal ion seems to play little or no role in the growth process of the PBA particles.

**Figure 10:**
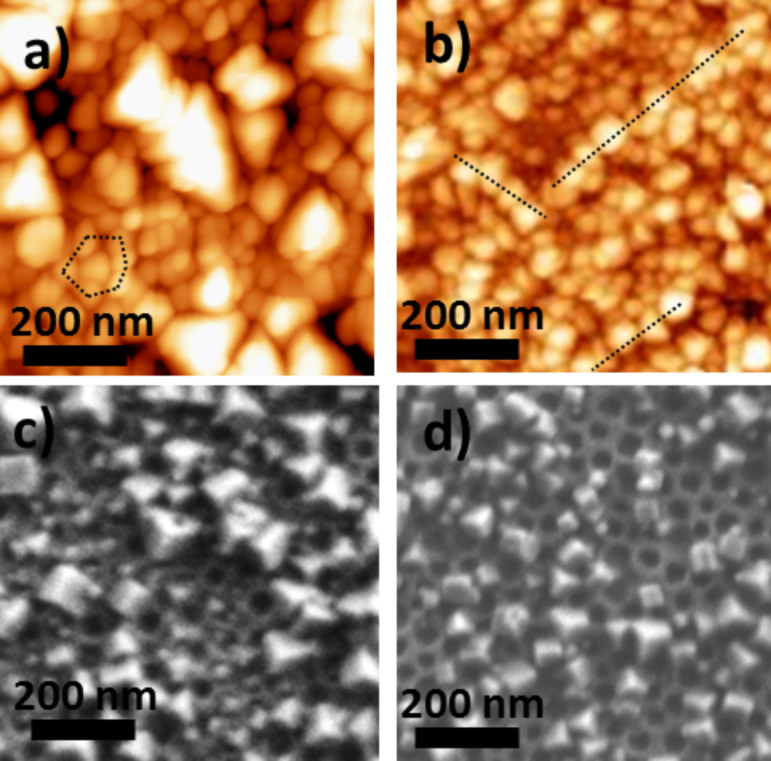
AFM images of a) **NC04**, b) **NC05** and SEM micrographs of c) **NC04**, d) **NC05**.

**Effect of the solvent or of the solvent mixture of the PBA precursors solutions**: The TiO_2_/CoFe PBA nanocomposites synthesized by using alcohol or a water/alcohol mixture for the PBA growth were studied by AFM and SEM. Figure 10a and Figure 10c show representative AFM and SEM images of the TiO_2_/CoFe PBA nanocomposite **NC04** synthesized by using a butanolic Co^2+^ solution and [Fe(CN)_6_]^3−^ in a 1:1 EtOH/H_2_O mixture as PBA precursors solutions. Figure 9b and Figure 9d show representative AFM and SEM images of the TiO_2_/CoFe PBA nanocomposite **NC05** synthesized by using solutions of Co^2+^ in a 1:1 EtOH/H_2_O mixture and [Fe(CN)_6_]^3−^ in 1:1 EtOH/H_2_O mixture as PBA precursors solutions. The AFM and SEM images are very different from those of the previous samples (**NC01** to **NC03**). This difference shows the important role of the reaction medium for PBA growth. The images show a great number of faceted particles with a pyramidal shape or a cubic shape with one corner inserted in one perforation.

Except some differences in the particles size range, which seems somewhat larger in the case of **NC04** than in the case of **NC05**, the AFM and SEM images of **NC04** and of **NC05** are very similar. The average density of filled perforations is very high for the two samples, which makes it difficult to see if the particles are localized in the perforations. However, some organization of the particles can be seen in the AFM images (dotted lines in Figure 10a and Figure 10b), which could result from the organization of the perforations. For instance, a hexagonal organization of some particles is visible and the distance between the aligned particles fairly corresponds to the distance between the centers of the perforations. **NC04** and **NC05** exhibit particles with a completely different shape compared to samples **NC01** to **NC03**. Because the particles have the same chemical composition (see IR spectroscopic study below), these different shapes probably reflect different determining factors in the particles formation. Indeed, different shapes for particles of same chemical composition usually correspond to growth rates limited by different parameters. PBA particles crystallize in the *Fm*−3*m* cubic face centered space group. They are often cubic due to different interface energies for the different crystallographic faces and therefore different growth rates leading to the formation of cubic particles. Nevertheless, under some specific conditions of concentrations, spherical particles can be obtained when the growth reaction rate is limited by the diffusion of the precursors. In the case of the films, the pyramidal or cubic shape is systematically obtained when the reaction medium is BuOH or the 1:1 mixture EtOH/H_2_O whereas the half-spherical or spherical shape is obtained in aqueous solutions. PBA particles are less soluble in BuOH and in the EtOH/H_2_O mixture than in H_2_O and therefore the dissolution–recrystallization rates are different. The diffusion rates of the precursors in the two reaction media are also different. These differences probably accounts for the different shape of the particles.

A pyramidal shape has been predicted by Tricard et al. [27–28] for a layer-by-layer growth process without additional dissolution–recrystallization phenomena. The study of the mechanisms involved in the PBA nanoparticles growth is still in progress and seems to be more complicated than the layer-by-layer process often evoked in the literature to describe the growth of PBA films [16–18].

#### Infrared spectroscopic investigation

Infrared spectroscopy, and especially the ν(C≡N) vibration band located in the spectral range of 2100–2200 cm^−1^ is usually used to characterize PBA species. Indeed, the cyanide bridge is extremely sensitive to its environment, including the oxidation state and the spin state of the transition metal ions [29].

**Figure 11:**
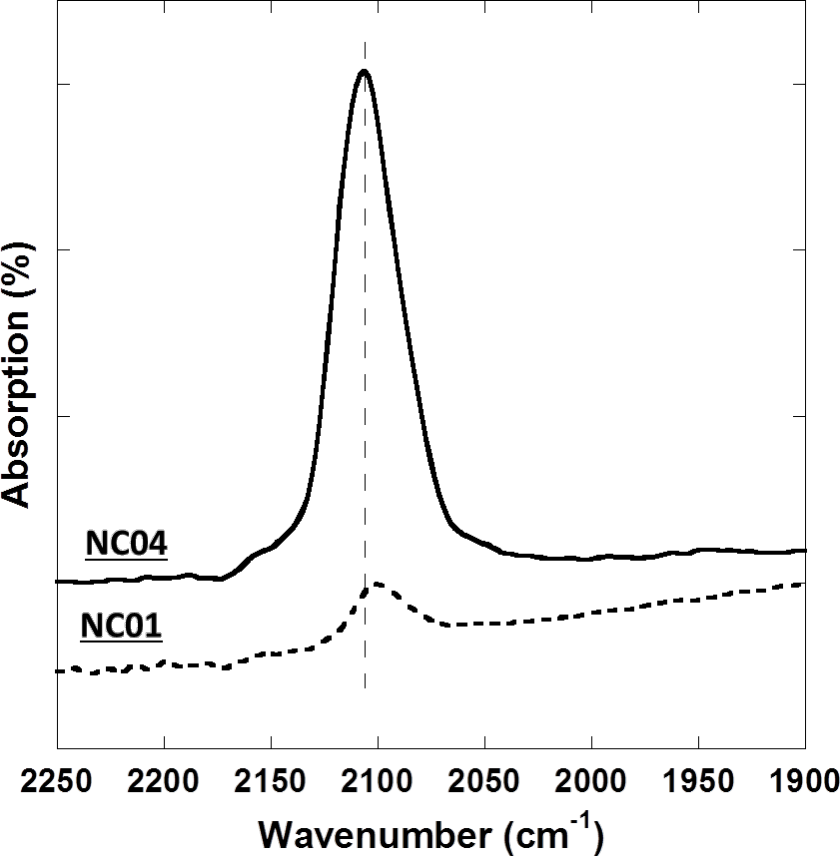
IR spectra of **NC01** and **NC04**.

Because samples **NC01** to **NC03** on the one hand and samples **NC04** and **NC05** on the other hand are very similar, the results are presented for samples **NC01** and **NC04**. The spectra of **NC01** and **NC04** are shown in the range of 1900–2250 cm^−1^ in Figure 11. Both spectra display one narrow and intense band centered at 2106 cm^−1^. This band is attributed to the CN stretching vibration. The position of the band corresponds neither to a terminal Fe^III^–CN group (expected around 2118 cm^−1^) [29] nor to a Fe^II^–CN group (expected around 2044 cm^−1^) [29]. The narrowness of this band and its energy position are in line with the formation of a CoFe PBA. The band is located at the same energy (within the experimental resolution) in both spectra. This indicates the formation of PBA of the same chemical composition for all the samples. The position of the band corresponds to CN in Fe^II^–CN–Co^II^ linkages of the Co^II^Fe^II^ PBA. Starting from Co^II^ and [Fe^III^(CN)_6_]^3−^ ions as PBA precursors, the energy position of the band indicates the reduction of [Fe^III^(CN)_6_]^3−^ into [Fe^II^(CN)_6_]^4−^ during the formation of PBA. Such a reduction reaction during the synthesis of PBA thin films has already been observed [30]. The strong difference in the intensity of the band from **NC04** to **NC01** reflects different amounts of PBA on the surface of the films. The intensity ratio of the bands of **NC04** and **NC01** is 15% in agreement with the average density of filled perforations determined by microsopy: around 15% for **NC01** and very high, close to 100%, for **NC04**.

## Conclusion

In conclusion, this first approach to study step-by-step the controlled growth of PBA particles within the perforations of ordered nanoperforated oxide layers allows for pointing out the steps the optimization of which would improve the final composite. Some steps are well-controlled or already optimized and some others are trickier and need to be optimized in order to get perfectly ordered nanoperforated layers with all the perforations filled with PBA particles.

After deposition, the small roughness of the gold layer is suitable for the subsequent deposition of the oxide layer. Nevertheless, the morphology of this layer undergoes important changes during the thermal treatment (step 3). The evolution is spectacular in the case of the 10 nm thick gold layer, but also exists for the layers with higher thicknesses. The unexpected thermal behavior of the gold layer shows that the first step clearly needs to be optimized to avoid any gold displacement modifying the planarity of the layer, which necessarily affects the ordered organization of the nanoperforations.

The deposition (step 2) and the thermal treatment (step 3) of the ordered hybrid organic–inorganic layer have already been extensively studied [12,19]. The smaller the diameters of the perforations are, the higher is the degree of order. Nevertheless, a perfect accessibility of the gold bottom of all the perforations is mandatory in order to fill all of them with PBA particles. Work is in progress to check and to improve this point.

The selective functionalization of the surfaces is a necessary step to localize PBA growth within the nanoperforations. The hydrophobization of the accessible oxide layer efficiently avoids the formation of PBA outside the perforations. The chemical nature of the anchoring function for the PBA growth seems not to be a determining factor in the average density of filled perforations.

The growth of PBA is undoubtedly the trickiest step. The average density of filled perforations and the shape of the particles seem strongly linked to the solubility of the growing particles in the reaction media. Work is in progress in order to fully control this step.

## Supporting Information

File 1 Additional experimental data.

## References

[1] Entley W R, Girolami G S (1995). Science.

[2] Ferlay S, Mallah T, Ouahes R, Veillet P, Verdaguer M (1995). Nature.

[3] Sato O, Iyoda T, Fujishima A, Hashimoto K (1996). Science.

[4] Bleuzen A, Lomenech C, Escax V, Villain F, Varret F, Cartier dit Moulin C, Verdaguer M (2000). J Am Chem Soc.

[5] Li D, Clérac R, Roubeau O, Harté E, Mathonière C, Le Bris R, Holmes S M (2008). J Am Chem Soc.

[6] Mercurol J, Li Y, Pardo E, Risset O, Seuleiman M, Rousselière H, Lescouëzec R, Julve M (2010). Chem Commun.

[7] Hoshino N, Iijima F, Newton G N, Yoshida N, Shiga T, Nojiri H, Nakao A, Kumai R, Murakami Y, Oshio H (2012). Nat Chem.

[8] Cafun J-D, Cartier dit Moulin C, Fornasieri G, Arrio M-A, Briois V, Bleuzen A (2011). New J Chem.

[9] Ludi A, Güdel H (1973). Structural chemistry of polynuclear transition metal cyanides. Inorganic Chemistry.

[10] Brinker C J, Scherer G W (1990). Sol-Gel Science: The Physics and Chemistry of Sol-Gel Processing.

[11] Kresge C T, Leonowicz M E, Roth W J, Vartuli J C, Beck J S (1992). Nature.

[12] Fisher A, Kuemmel M, Järn M, Linden M, Boissière C, Nicole L, Sanchez C, Grosso D (2006). Small.

[13] Schulze C, Faustini M, Lee J, Schletter H, Lutz M U, Krone P, Gass M, Sader K, Bleloch A L, Hietschold M (2010). Nanotechnology.

[14] Allouche J, Lantiat D, Kuemmel M, Faustini M, Laberty C, Chaneac C, Tronc E, Boissiere C, Nicole L, Sanchez C (2010). J Sol-Gel Sci Technol.

[15] Lepoutre S, Grosso D, Sanchez C, Fornasieri G, Rivière E, Bleuzen A (2010). Adv Mater.

[16] Volatron F, Heurtaux D, Catala L, Mathonière C, Gloter A, Stéphan O, Repetto D, Clemente-León M, Coronado E, Mallah T (2011). Chem Commun.

[17] Pajerowski D M, Gardner J E, Frye F A, Andrus M J, Dumont M F, Knowles E S, Meisel M W, Talham D R (2011). Chem Mater.

[18] Cobo S, Molnár G, Carcenac F, Szilágyi P Á, Salmon L, Vieu C, Bousseksou A (2010). J Nanosci Nanotechnol.

[19] Kuemmel M, Allouche J, Nicole L, Boissière C, Laberty C, Amenitsch H, Sanchez C, Grosso D (2007). Chem Mater.

[20] Mutin P H, Guerrero G, Vioux A (2003). C R Chim.

[21] Guerrero G, Mutin P H, Vioux A (2001). Chem Mater.

[22] Sanchez C, Boissière C, Grosso D, Laberty C, Nicole L (2008). Chem Mater.

[23] Faustini M, Drisko G L, Boissiere C, Grosso D (2014). Scr Mater.

[24] Faustini M, Boissière C, Nicole L, Grosso D (2014). Chem Mater.

[25] Shon Y-S, Gross S M, Dawson B, Porter M, Murray R W (2000). Langmuir.

[26] Pasquinet E, Bouvier C, Thery-Merland F, Hairault L, Lebret B, Méthivier C, Pradier C M (2004). J Colloid Interface Sci.

[27] Tricard S, Raza Y, Mazerat S, Aissou K, Baron T, Mallah T (2013). Dalton Trans.

[28] Tricard S, Fabrice C, Mallah T (2013). Dalton Trans.

[29] Nakamoto K (1986). Infrared and Raman spectra of inorganic and coordination compounds.

[30] Bonhommeau S, Pontius N, Cobo S, Salmon L, de Groot F M F, Molnár G, Bousseksou A, Durr H A, Eberhardt W (2008). Phys Chem Chem Phys.

