# Supplementary material for: Towards bottom-up nanopatterning of Prussian blue analogues
Source: Beilstein J Nanotechnol. 2014 Oct 31;5:1933–43. doi: 10.3762/bjnano.5.204 (PMC4222400; doi:10.3762/bjnano.5.204)
Supplement: File 1 — Additional experimental data. [file Beilstein_J_Nanotechnol-05-1933-s001.pdf]

# Supporting Information

for

# Towards bottom-up nanopatterning of Prussian blue analogues

Virgile Trannoy[1], Marco Faustini[2], David Grosso[2], Sandra Mazerat[1], François Brisset[1], Alexandre Dazzi[3] and Anne Bleuzen*[1]

Address: [1]Institut de Chimie Moléculaire et des Matériaux d'Orsay,UMR CNRS 8182, Université Paris-Sud, 15 rue Georges Clémenceau, 91405 Orsay Cedex, France, [2]Laboratoire de Chimie de la Matière Condensée de Paris, Université Pierre et Marie Curie-Paris 6 and CNRS Collège de France, 11 place Berthelot 75231 Paris, France and [3]Laboratoire de Chimie Physique, UMR CNRS 8000, Université Paris-Sud, 15 avenue Jen Perrin, 91405 Orsay Cedex, France

Email: Anne Bleuzen* - anne.bleuzen@u-psud.fr

* Corresponding author

## Additional experimental data

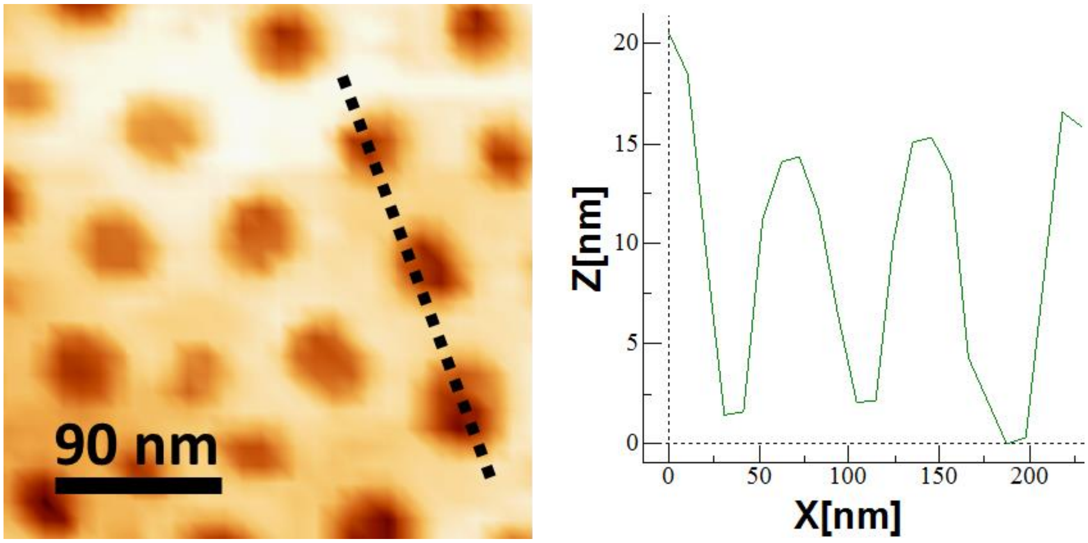


**Figure S1:** AFM image in a dark area of the sample **Au10NC** and height profile along the dotted line. The height maxima correspond to the top of the $TiO_2$ film and the minima to the bottom of the craters. The height of the film is around 15 nm. The distance between two adjacent perforations is around 80 nm.

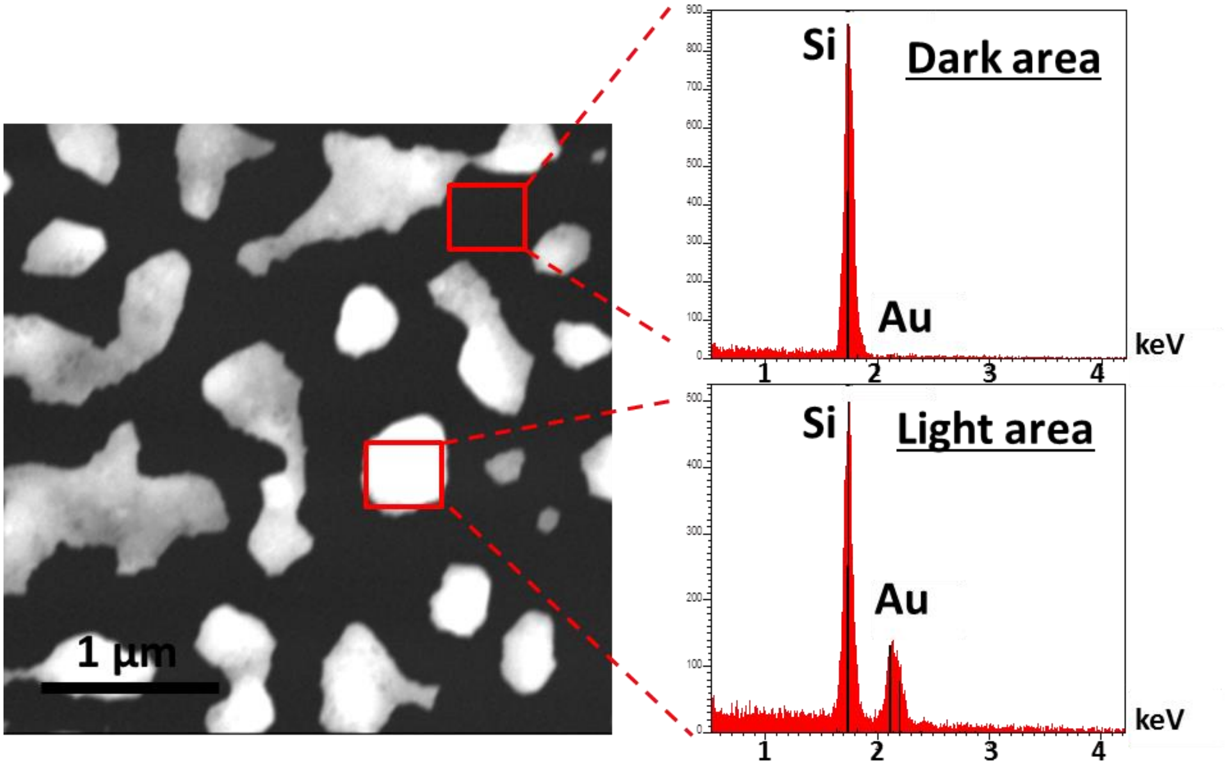


**Figure S2:** SEM image of the sample **Au10NC** and EDX analyses in a dark area and in a light area (No band corresponding to the Ti element is expected on this energy range).

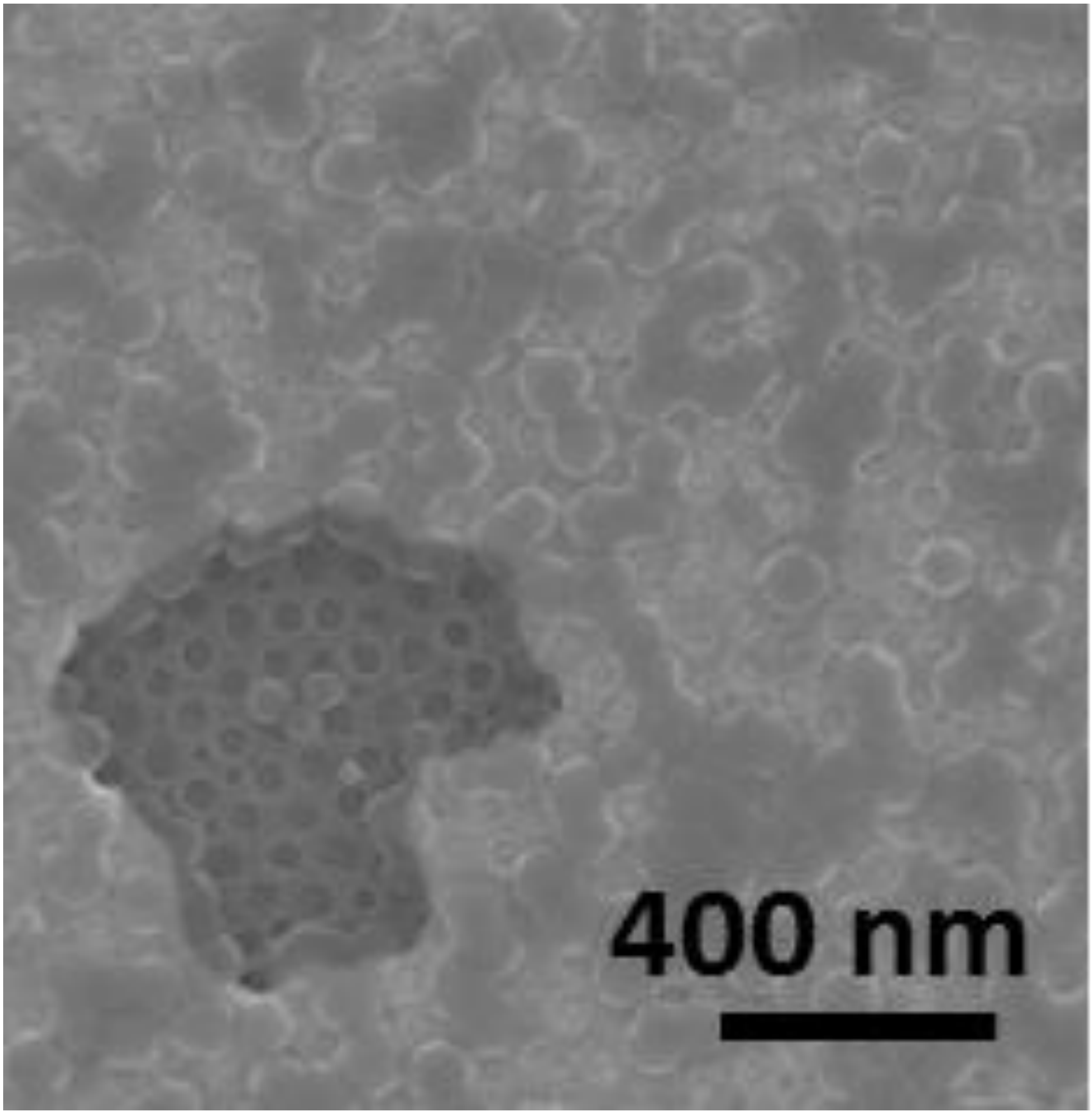


**Figure S3:** SEM images of a $TiO_2$/PBA nanocomposite perform with an in-Lens detector working at 5 kV and at a short working distance (WD) equal to 2.7 mm. PBA particles appear as protuberances sticking out of the nanoperforations in the bright zone containing the gold layer.
